# Unilateral Vibration Stimulation in Patients With Post-stroke Spasticity Suppresses Muscle Tonus in the Contralateral Homologous Muscles

**DOI:** 10.7759/cureus.91360

**Published:** 2025-08-31

**Authors:** Kenta Kunoh, Takahiro Takenaka, Daisuke Kimura, Toshiaki Suzuki

**Affiliations:** 1 Department of Health Sciences, Graduate School of Health Sciences, Kansai University of Health Sciences, Osaka, JPN; 2 Department of Rehabilitation, Yamada Hospital, Gifu, JPN; 3 Department of Rehabilitation, Heisei College of Health Sciences, Gifu, JPN; 4 Division of Occupational Therapy, Naragakuen University, Nara, JPN

**Keywords:** h-reflex, muscle stiffness, spasticity, stroke, vibration

## Abstract

Objective: This study aimed to examine whether unilateral vibration stimulation can reduce spasticity during the stimulation period in post-stroke patients.

Methods: Ten post-stroke patients with increased muscle tone in the flexor carpi radialis (FCR) participated. Vibration stimulation at 80 Hz was applied to the paretic-side FCR. The H-reflex and muscle stiffness were assessed before, during, and immediately after stimulation. Changes in H-reflex parameters were analyzed alongside muscle stiffness using the Wilcoxon signed-rank test.

Results: H-reflex measurements showed a temporary reduction in spasticity during stimulation. However, no significant change was observed in muscle stiffness.

Conclusion: Unilateral vibration stimulation may offer immediate neurophysiological suppression of spasticity during application, although it does not appear to affect muscle stiffness in the short term. These findings suggest potential for use as a complementary intervention in stroke rehabilitation.

## Introduction

One of the clinical symptoms of stroke is spasticity. Spasticity is defined as an increase in speed-dependent stretch reflexes and is observed in approximately 17%-25% of stroke patients [1], making it a symptom that receives significant attention in rehabilitation settings. Furthermore, spasticity has been reported to have a significant impact on activities of daily living (ADL) and quality of life (QOL) [2]. Specifically, spasticity is known to easily affect voluntary movements of the wrist and fingers [3], and in ADL, it has been reported that difficulty in opening the fingers can lead to nail ingrowth and pain [4]. In terms of QOL, the degree of mobility, self-care, work and leisure, pain and discomfort, and anxiety and depression are considered to affect health-related QOL [2]. Based on the above, it can be said that there is a certain significance in taking effective measures against spasticity.

Against this background, numerous rehabilitation approaches for spasticity have been investigated. A review of previous studies indicates that most rehabilitation approaches for spasticity are considered effective, with physical therapy being widely used, including electrical stimulation therapy and thermotherapy. Furthermore, among physical therapies, vibration stimulation therapy is considered a representative rehabilitation intervention for spasticity due to its ability to provide inexpensive and reproducible stimulation [5-7]. The neurophysiological mechanism underlying the intervention effect of vibration stimulation on spasticity is thought to involve the excitation of muscle spindles on the vibration stimulation side, which transmits sensory information via Ia fibers [8,9]. Sensory information via these Ia fibers is thought to suppress muscle tonus through two mechanisms. First, there is a method that promotes post-activation depression by directly stimulating the affected muscles. This is a polysynaptic reflex of Ia fibers, which is considered a method of suppressing increased muscle tone by promoting depletion of neurotransmitters and changes in muscle spindle sensitivity [10-12]. The next method involves stimulating the antagonist muscles of the affected muscles. This method promotes the tonic vibration reflex, which induces a monosynaptic reflex via Ia fibers in the vibrated muscle, acting as a facilitatory stimulus on the stimulated muscle and promoting reciprocal inhibition of its antagonist, the affected muscle [13]. However, it has been clearly demonstrated that vibration stimulation does not produce long-term effects because the muscle tonus-inhibiting effect is lost after the stimulation ends [14-16].

Based on the above, the relationship between vibration stimulation and exercise therapy in spasticity can be summarized as follows: vibration stimulation plays a role in suppressing muscle tonus during stimulation. However, the effect lasts only during vibration stimulation and does not persist after vibration stimulation, and therefore, no long-term effect was observed. Furthermore, in rehabilitation settings, we often observe that muscle tonus immediately increases when vibration stimulation is used to suppress muscle tonus, followed by exercise therapy aimed at improving the voluntary control of the antagonist muscles. This suggests that it is not appropriate to use vibration stimulation to the wrist and fingers separately from exercise therapy and that combining vibration stimulation aimed at suppressing muscle tonus with exercise therapy may yield high efficacy. One method for achieving this is to stimulate the contralateral homologous muscle corresponding to the affected muscle, and its effectiveness has been demonstrated in previous studies [17-19]. The advantages of applying this method to rehabilitation interventions in clinical settings include the ability to prevent movement of the paralyzed side, simultaneously provide vibration stimulation to the non-paralyzed side and exercise therapy to the paralyzed side, and potentially suppress muscle tonus during movement. In other words, it can be said that the effects of vibration stimulation, which has been used before and after exercise therapy to suppress muscle tonus, may be extended to include the suppression of muscle tonus during exercise. However, there has been insufficient investigation into whether the desired effects can be achieved in patients with spasticity, and this remains an issue. Unilateral vibration stimulation has been suggested to potentially reduce spasticity. This intervention may serve as an effective adjunct to exercise therapy, helping to control muscle tone during upper limb movements in stroke patients with spasticity. Therefore, the purpose of this study was to investigate whether unilateral vibration stimulation suppresses spasticity during the stimulation period in patients with post-stroke spasticity. Spasticity was evaluated using neurophysiological measures such as the H-reflex and mechanical properties including muscle stiffness to capture both neural and structural components. This study also focused on the effects of unilateral stimulation on the contralateral homologous muscle to examine its influence across sides. We hypothesized that unilateral vibration would reduce spinal excitability and thereby decrease spasticity in the contralateral limb during stimulation.

## Materials and methods

Participants

This study employed a single-group pre-post intervention design to examine the immediate effects of unilateral vibration stimulation on spasticity in post-stroke patients. This study was conducted at Yamada Hospital in Gifu, Japan, between January and May 2025. The study included 10 patients (age 65.6 ± 15.9 years) who had spasticity after a stroke. Prior to participant recruitment, an a priori power analysis was conducted using G*Power software (version 3.1.9.7; Heinrich-Heine-Universität Düsseldorf, Düsseldorf, Germany) to estimate the required sample size. Based on a two-tailed Wilcoxon signed-rank test for two time points in matched pairs, assuming a large effect size (dz = 0.66), an alpha level of 0.05, and a statistical power of 0.80 (1-β), the minimum required sample size was calculated to be 21. However, due to recruitment limitations and the exploratory nature of this study, 10 participants were ultimately enrolled. In the present study, several clinical assessments were administered to characterize the overall features of the participants. Spasticity was assessed using the Modified Ashworth Scale (MAS), which evaluates the resistance encountered in a muscle during passive joint stretching and allows for an objective assessment of spasticity [20]. Cognitive function was evaluated using the Hasegawa Dementia Scale Revised (HDS-R), a nine-item screening tool for dementia. The HDS-R is characterized by its reduced susceptibility to individual differences compared with other dementia screening instruments [21]. Finally, upper limb motor function was assessed using the upper extremity section of the Fugl-Meyer Assessment (FMA) [22], which comprises four subdomains and provides a maximum score of 66 points. Inclusion criteria were upper limb motor impairment after a stroke and a MAS score of 1 or higher for the flexor carpi radialis muscles (FCR). The MAS has been widely used as a clinical tool for assessing post-stroke spasticity and has demonstrated sufficient inter-rater reliability [20,23]. Exclusion criteria were as follows: participants with an FCR MAS score of 4, those unable to maintain the starting position of the examination due to joint range of motion limitations, those with upper limb motor disorders not caused by stroke, those with bilateral hemiplegia, those with an HDS-R score of 21 or lower, and those with superficial or deep sensory loss. All the participants received verbal and written explanations of the study content and ethical considerations to ensure adequate understanding, including the risks and freedom to participate, before enrollment. Written informed consent was obtained from all participants. The study conformed to the guidelines of the Declaration of Helsinki. The Research Ethics Review Committee of Kansai University of Health Sciences, Osaka, Japan, approved the study (approval number: 24-31, 21-10-2024) before initiation of the protocol. This study was also registered with the University Hospital Medical Information Network Clinical Trials Registry (UMIN-CTR) (Registration No. UMIN000058736).

H-reflex recording

**Figure 1:**
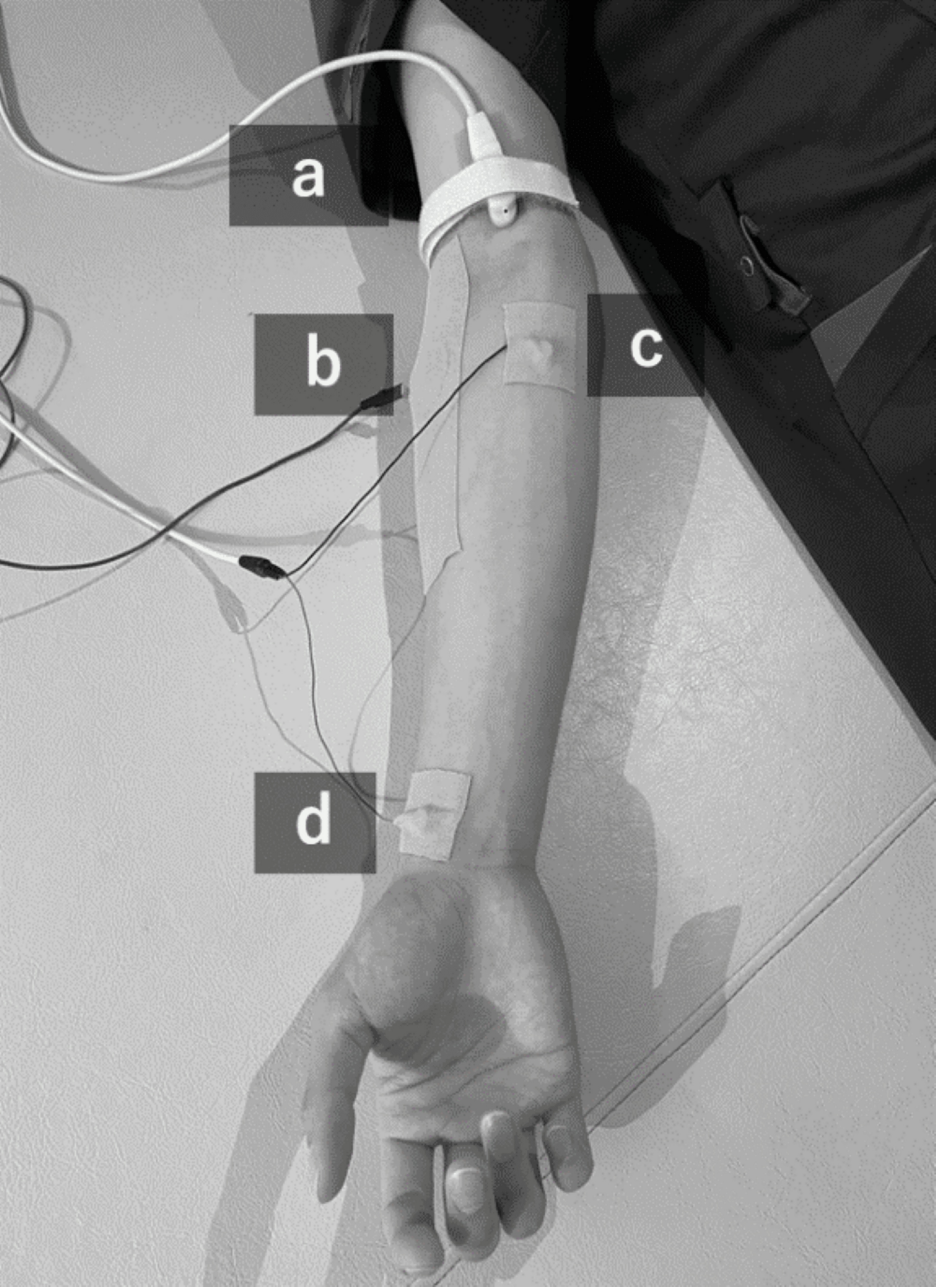
Electrode placement for H-reflex measurement a: Stimulation electrode, b: plate ground electrode, c: exploration electrode, and d: reference electrode.

In this study, H-reflex was used to objectively evaluate spasticity. H-reflex is an action potential obtained from the muscles under innervation by excitatory stimulation of the afferent Ia fibers of the stretch reflex. The conditions for measuring the H-reflex were as follows: the equipment used was Neuropack S3 (Nihon Kohden Co., Ltd., Tokyo, Japan). The measurement position was supine with the subject at rest, and the test muscle was the FCR on the paralyzed side. The forearm on the paralyzed side was in a supinated position. Regarding the recording conditions for the H-reflex, the probe electrode was placed on the FCR at the proximal one-third of the line connecting the medial epicondyle to the radial styloid process, and the reference electrode was placed on the radial styloid process [24,25] (Figure 1).

The ground electrode was attached to the lateral side of the forearm. In addition, the frequency band was set to 10 Hz to 5 kHz, and the sampling frequency was set to 1 kHz. Furthermore, regarding the H-reflex stimulation conditions, the stimulation site was set to the median nerve at the elbow joint. The stimulation intensity was set to 120% of the M-wave threshold, the stimulation frequency was set to 0.1 Hz, the stimulation duration was set to 1.0 ms, and the number of stimulations was set to eight consecutive stimulations. The evaluation indices used for analyzing the H-reflex waveform were the H/M amplitude ratio and the mean amplitude. The H-reflex was measured before vibration stimulation and immediately after the start of vibration stimulation, and vibration stimulation was continuously applied during the H-reflex measurement.

Muscle stiffness measurement

To quantitatively evaluate changes in muscle tonus, a muscle stiffness meter was used. The device employed was the NEUTONE TDM-Z2 muscle hardness meter (Try-all Co., Ltd., Chiba, Japan), which has been widely used in previous studies [26,27]. This device measures the reaction force exerted by a muscle when pressure is applied and converts the value into Newtons (N) using a manufacturer-specified formula. The muscle evaluated for stiffness was the flexor carpi radialis (FCR) on the paretic side. The measurement site was located 3 cm proximal to the electrode used for H-reflex recording. Muscle stiffness was measured five times at each time point, and the average was used for analysis. Measurements were taken at two time points: (1) immediately before the start of vibration stimulation (pre-vibration), and (2) during the vibration stimulation, immediately following the H-reflex measurement (during-vibration).

Vibration stimulation conditions

**Figure 2:**
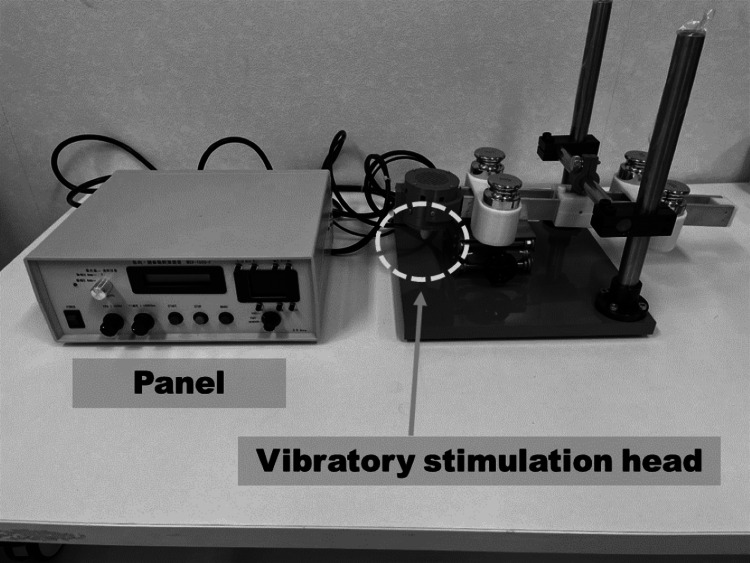
Vibration stimulator used in this study The vibratory stimulation head is the part that provides the vibration stimulus. The panel allows you to adjust the frequency, amplitude and stimulus time.

Vibration stimulation generally requires setting the frequency, amplitude, load weight, stimulation site, and stimulation time [15,28]. In this study, a vibration stimulus with a frequency of 80 Hz, amplitude 0.4 mm, and load weight 400 g was applied for 70 seconds to the FCR on the paralyzed side, while the participants were in a resting supine position [29-33]. A muscle-tendon vibratory stimulation device, MGV-1000-F (Uchida Electron Co., Ltd., Tokyo, Japan), was utilized to apply the vibratory stimulation (Figure 2).

Statistical analysis

In the statistical analysis, the Shapiro-Wilk test was performed to assess normality. As the assumption of normality was not met, comparisons of the H/M amplitude ratio, mean amplitude, and muscle stiffness between before and during stimulation were conducted using the Wilcoxon signed-rank test. Due to the small sample size, effect sizes (r) were calculated to support the interpretation of the results. All analyses were performed using SPSS Statistics version 30.0 (IBM Corp., Armonk, NY, USA), and the significance level was set at 5%.

## Results

The basic characteristics of the subjects in this study are shown in Table 1. The study sample consisted of six male and four female participants. The mean time since stroke onset was 9.9 ± 10.9 months, corresponding to the subacute to chronic phase. The affected side was the right in four participants and the left in six. The lesion locations in each participant corresponded to brain regions commonly associated with the development of spasticity. The mean Fugl-Meyer Assessment (FMA) score was 14.8 ± 12.5, indicating a wide range of motor impairment severity from severe to mild. Additionally, all participants had Modified Ashworth Scale (MAS) scores of 1, 1+, or 2.

**Table 1:** Participant characteristics FMA-UE: Fugl-Meyer Assessment for the Upper Extremity, MAS: Modified Ashworth Scale.

Patients	Age	Gender	Months Since Stroke	Side	CT/MRI	FMA-UE	MAS
1	89	Female	3	Right	Corona radiata	24	1
2	59	Male	6	Left	Medulla oblongata	6	1+
3	43	Male	25	Left	Putamen	14	1+
4	61	Female	2	Right	Thalamus	21	1+
5	51	Male	36	Left	Putamen	13	2
6	55	Female	8	Left	Frontoparietal cortex	46	1+
7	85	Female	3	Right	Corona radiata	2	1
8	82	Male	3	Left	Corona radiata	3	1+
9	51	Male	11	Right	Putamen	13	2
10	80	Male	2	Left	Putamen	6	1

**Figure 3:**
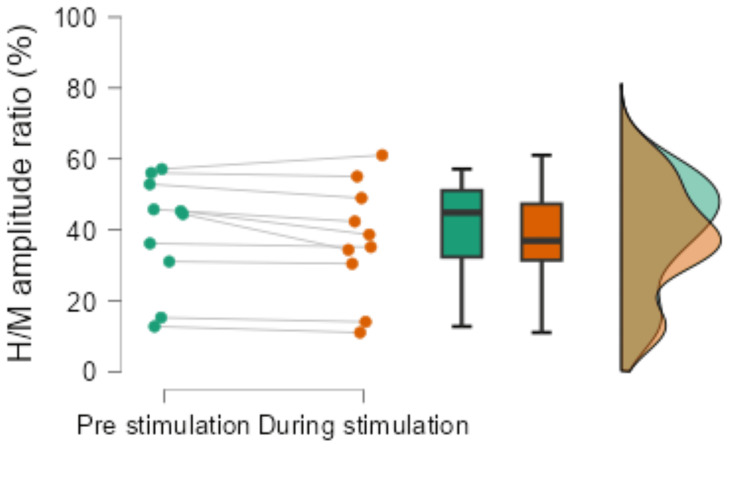
Results of H/M amplitude ratio before and during vibration stimulation

The H/M amplitude ratio pre-stimulation was 44.9 ± 15.8%, and during stimulation, it was 36.9 ± 16.1%, showing a significantly lower value during stimulation (p = 0.041, r = -0.65, Figure 3).

**Figure 4:**
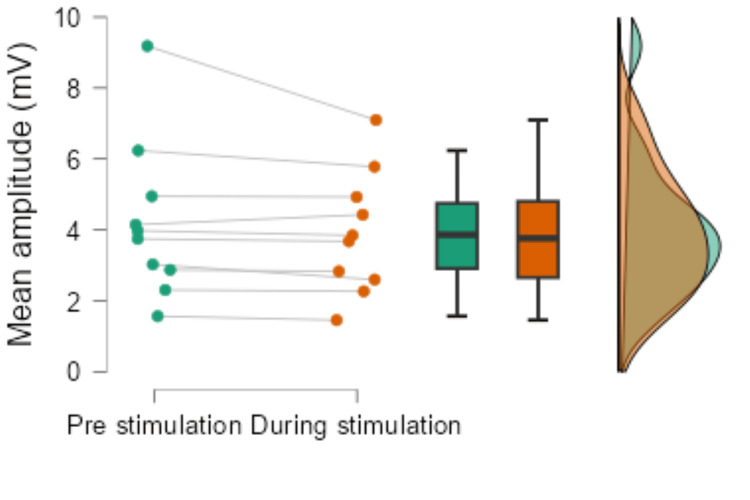
Results of H-reflex mean amplitude before and during vibration stimulation

The mean amplitude during pre-stimulation was 3.87 ± 2.20 mV, and the mean amplitude during stimulation was 3.76 ± 1.72 mV, showing a significant decrease during stimulation (p = 0.037, r = -0.66, Figure 4). These reductions in H/M ratio and mean amplitude indicate a decreased excitability of spinal reflexes during stimulation, serving as important objective markers of spasticity reduction.

**Figure 5:**
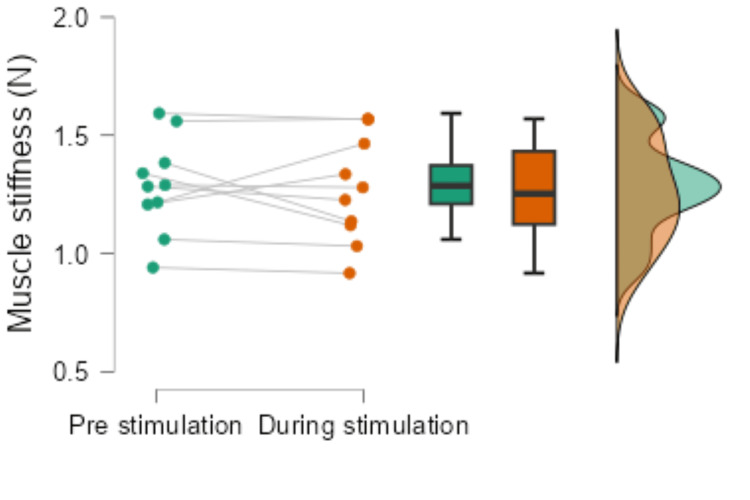
Results of muscle stiffness before and during vibration stimulation

Muscle stimulation during pre-stimulation was 1.29 ± 0.20 N, and muscle stiffness during stimulation was 1.25 ± 0.22 N, with no significant difference (p = 0.359, r = -0.29, Figure 5). The lack of change in muscle stiffness suggests that short-term vibration stimulation may not alter the mechanical properties of the muscle.

## Discussion

In summary, significant differences were observed in the H/M amplitude ratio and H-reflex mean amplitude, but no significant differences were observed in muscle stiffness. Therefore, the effectiveness of the non-paretic side for spasticity is discussed below.

Neural mechanisms of crossed inhibitory effects induced by unilateral vibratory stimulation

In this result, the H/M amplitude ratio and mean amplitude were significantly lower during stimulation. Previous studies have shown that vibratory stimulation of the abductor pollicis brevis muscle in healthy individuals contributes to a decrease in the excitability of spinal anterior horn cells corresponding to the contralateral homologous muscle [18]. Furthermore, in healthy individuals, it has been demonstrated that stimulating the tibial nerve with a constant stimulus intensity using H-reflex results in the inhibition of H-reflexes derived from the soleus muscles on the contralateral side. This suggests that sensory information transmitted via Ia fibers contributes to the inhibition of muscle tone in the contralateral homologous muscle via interneurons in the spinal cord [34,35]. From the above, it is suggested that the neural mechanism mediated by interconnecting neurons may function commonly in both the upper and lower limbs. However, it is unclear whether the same neural mechanisms function in stroke patients. On the other hand, regarding the involvement of higher centers, previous studies have reported that in healthy individuals, both primary motor cortices may be activated when vibration stimuli are applied to one side. However, in stroke patients, when vibration stimuli are applied to the paralyzed side, activity in the primary motor cortex controlling the paralyzed side is observed unilaterally [36]. Therefore, these results support the notion that, in stroke patients as well as in healthy individuals, the spinal cord contributes to the inhibition of muscle tone on the contralateral side.

Effects of vibratory stimulation to the non-paretic side from the perspective of primary and secondary impairments in spasticity

In this study, although the H/M amplitude ratio and mean amplitude showed significantly lower values during stimulation, no significant difference was observed in muscle stiffness. Regarding this matter, spasticity has traditionally been divided into two categories: primary disorders, which are known to be essential mechanisms involving speed-dependent stretch reflex enhancement, and secondary disorders, which represent changes in muscle characteristics, such as muscle shortening, caused by increased muscle tonus due to stretch reflex enhancement. In a previous study, experts from 12 countries conducted an online survey and reached a consensus that interventions should be differentiated between primary and secondary disorders [37]. Furthermore, the H-reflex, which was used as an evaluation index in this study, is said to reflect the degree of spasticity [38]. Furthermore, during muscle hardness measurements in this study, the limbs were maintained in a supine position at rest, and care was taken to ensure that no changes in muscle stretching speed occurred during FCR. In other words, the aforementioned environment did not include factors that could cause an increase in muscle tension due to major diseases. In other words, the environment was not a condition that promoted an increase in muscle tension due to major diseases. Therefore, muscle stiffness is interpreted as a state that more readily reflects the effects of secondary disorders such as muscle shortening resulting from increased muscle tonus, rather than increased muscle tonus caused by primary disorders. Furthermore, when confirming the MAS of the subjects in this study, all subjects fell into one of the following categories: 1, 1+, or 2. MAS1 is defined as “a sensation of catching when the affected area is stretched, which disappears immediately afterward” or “a slight resistance at the end of the range of motion” [39,40]. Regarding this, the former reflects primary impairment in spasticity, while the latter reflects secondary impairment, and it can be interpreted that patients with MAS1 have either primary or secondary impairment. MAS1+ is defined as “a slight increase in muscle tonus demonstrated by stretching the affected area, followed by minimal resistance throughout the entire range of motion” [39,40]. Of these, the increase in muscle tonus in the first half reflects primary impairment, while the sustained resistance in the latter half reflects secondary impairment. Therefore, MAS1+ is a condition in which primary and secondary disorders coexist, but it is possible to distinguish between them in the evaluation. MAS2 is defined as “muscle tonus increases significantly throughout most of the range of motion, but the affected area can be moved easily” [39,40]. This condition can also be interpreted as a mixture of primary and secondary impairments, but it is difficult to clearly distinguish between the two in the assessment. In other words, the more severe the spasticity, the more likely it is that secondary impairments will overlap with primary impairments. In this study, while the primary disorder was reflected in the H-reflex, the secondary disorder was reflected in muscle stiffness. The significant decrease in the H-reflex-related indicators derived from the paralyzed side during stimulation of the non-paralyzed side suggests an inhibitory effect on the primary disorder, namely the speed-dependent stretch reflex at the spinal cord level. On the other hand, no significant changes were observed in muscle stiffness, suggesting that the effects on secondary disorders such as muscle shortening and structural changes in soft tissues are limited.

Clinical implications

Based on the above, for clinical application, vibratory stimulation of the non-paralyzed side is expected to immediately reduce spasticity on the paralyzed side in patients with MAS1 who primarily exhibit spasticity due to primary impairments. However, in patients with MAS1+ or MAS2, immediate effects may be enhanced by combining vibratory stimulation of the non-paralyzed side with rehabilitation interventions targeting secondary impairments on the paralyzed side.

Study limitations

Finally, there are several limitations to this study. First, the number of subjects was small, and the study was conducted at a single facility. In the future, it will be necessary to examine the reproducibility and generalizability of the results through multi-center collaborative research and expansion of the study population. Additionally, most participants had mild spasticity (MAS scores ranging from 1 to 2), which may limit the generalizability of the results to patients with moderate to severe spasticity. Future studies should include a wider range of spasticity severity. Second, this study evaluated only immediate effects and did not examine whether sustained spasticity suppression effects could be obtained. It is necessary to evaluate the long-term effects and the sustained effects of combining exercise therapy with other treatments. Third, this study did not include a control group or sham stimulation, which limits the ability to exclude placebo or non-specific effects. Fourth, neither participants nor assessors were blinded, which may introduce bias in outcome measurement. Fifth, functional outcome measures such as hand function or activities of daily living were not assessed, limiting clinical interpretability. Given the small sample size and preliminary nature of this study, the findings should be considered exploratory and interpreted with caution. Further large-scale, controlled studies are needed to confirm these results. Finally, clinical claims should be made cautiously, and the study should be positioned as an exploratory or pilot study due to these limitations.

## Conclusions

In this study, we investigated the immediate effects of vibration stimulation applied to the non-paralyzed side on spasticity in the homologous muscles of the paralyzed side in patients with hemiplegia and spasticity following stroke, using H-reflex and muscle stiffness. The results showed that vibration stimulation of the non-paralyzed side FCR significantly decreased the H/M amplitude ratio and mean amplitude on the paralyzed side, suggesting an immediate suppression of spinal reflex excitability, which corresponds to primary impairment in spasticity. On the other hand, no significant changes were observed in muscle stiffness, indicating that the immediate effects on the mechanical properties of peripheral muscle tissue that reflect secondary impairments may be limited. These results are considered to be immediately applicable to post-stroke patients with spasticity in whom primary impairments predominate. Caution is warranted when applying these findings to patients with more severe spasticity, where secondary impairments are more pronounced.
